# Osteopathic manipulative treatment as an adjunct therapy in a pediatric patient with chronic pancreatitis and visceral hyperalgesia: a case report

**DOI:** 10.3389/fmed.2026.1839129

**Published:** 2026-10-09

**Authors:** Avrohom Levy, Haili Bach, Fernando Medina Carbonell, Aniruddh Setya

**Affiliations:** Pediatric Gastroenterology Division, Department of Pediatrics, Saint Louis University School of Medicine SSM Health Cardinal Glennon Children’s Hospital, Saint Louis, MO, United States

**Keywords:** case report, central sensitization, gastroenterology, osteopathic manipulative treatment, pediatric gastroenterology, visceral hyperalgesia

## Abstract

**Background:**

Chronic abdominal pain arising from visceral hyperalgesia is difficult to manage, and the use of osteopathic manipulative medicine in pancreatitis-associated visceral hyperalgesia is rarely described. This case adds to the literature by highlighting a potential integrative, mechanism-informed approach for pain modulation in a complex pediatric patient.

**Case:**

A 17-year-old male with chronic pancreatitis in the setting of a heterozygous SPINK1 N34S (c.101A > G; p.Asn34Ser) variant, presented with acute-on-chronic abdominal pain severe enough to require escalating opioid therapy; imaging demonstrated calcifications, edema, and peripancreatic fluid collections. A multimodal treatment plan was initiated that included gabapentin, nortriptyline, and targeted osteopathic manipulative treatment. A secretin-enhanced MRCP obtained during a prior admission one month earlier demonstrated pancreatic calcifications, edema, and peripancreatic fluid collections. During the current hospitalization, serum lipase decreased from 979 U/L on admission to 90 U/L by hospital day 3, when gabapentin, nortriptyline, and targeted osteopathic manipulative treatment were initiated as part of a multimodal treatment plan. The patient had two sessions of osteopathic treatment and they were each associated with reductions in pain without adverse effects. As-needed opioid medications were discontinued by discharge as part of a multimodal therapy plan. The patient remained pain-free at one-month follow-up with minimal opioid use, suggesting a potential role for osteopathic manipulative medicine as part of a multimodal strategy in the treatment of chronic pancreatitis related pain. At three-month follow-up, the patient reported pain rated 4/10 occurring several times per week and subsequently underwent total pancreatectomy with islet autotransplantation for definitive management.

**Conclusion:**

This case describes an association between OMT and short-term pain improvement within a multimodal treatment strategy for chronic pancreatitis-related visceral pain. Although causality cannot be established from a single uncontrolled observation, the findings support further investigation of OMT as a potential adjunctive therapy in pediatric patients with chronic pancreatitis and secondary visceral hyperalgesia.

## 1 Introduction

Chronic pancreatitis is frequently associated with persistent abdominal pain that may become amplified through mechanisms of central sensitization and secondary visceral hyperalgesia. Pain in the setting of chronic pancreatitis is very common, with pain being present in more than 80% of chronic pancreatitis patients at some point during their clinical course, both for adults and children (1, 2). There are currently no medical therapies proven to prevent progression or recurrence of chronic pancreatitis, and treatment is largely directed toward symptom control and management of complications. For acute and chronic pain, medical therapies include analgesic agents, antioxidants, and neuromodulators. Opioids are also classically used, but their use should be limited due to risks of tolerance, addiction, narcotic bowel syndrome, and a paradoxical increase in pain due to opioid-induced hyperalgesia (3). When pain persists despite medical and endoscopic therapies and significantly impairs quality of life, surgical intervention may be considered. Surgical approaches include drainage procedures to decompress a dilated pancreatic duct, pancreatic resections, or combined drainage-resection operations. Total pancreatectomy with islet autotransplantation (TPIAT) is an option for selected patients who lack suitable anatomy for drainage procedures or partial resection, have persistent symptoms despite conventional surgical approaches, or have underlying genetic risk factors associated with progressive disease (4). However, pain may persist in a subset of patients even after TPIAT, suggesting that factors beyond ongoing pancreatic inflammation contribute to symptom burden (5).

Additional interventions for refractory chronic pancreatitis-related pain include celiac plexus and splanchnic nerve blocks, which may be performed under fluoroscopic, computed tomography, or endoscopic ultrasound guidance, as well as vagal nerve stimulation. These approaches are based on the neural anatomy of the pancreas, which receives sympathetic innervation through the splanchnic nerves and celiac plexus as well as parasympathetic innervation through the vagus nerve (6).

Despite advances in pain management, chronic pancreatitis-associated pain remains challenging to treat, and a subset of patients continue to experience significant symptoms despite therapy. This persistent pain after therapy may reflect the contribution of neuropathic pain and central sensitization (2), but highlights the need for addition adjunctive approaches.

Osteopathic manipulative medicine uses hands-on diagnosis and treatment to influence autonomic input to the gastrointestinal system. Palpable tissue texture changes in predictable body regions can indicate visceral GI dysfunction, and targeted osteopathic manipulative therapy (OMT) has been proposed to influence viscero-somatic or viscero-visceral reflexes to interrupt maladaptive feedback. These interventions are thought to reduce nociceptive facilitation at spinal or visceral levels and help restore autonomic balance (7). Considering the neural anatomy of the pancreas, adding OMT as a complementary, mechanism-based approach could expand options and address unmet needs. As a hands-on, low-risk integrative therapy, OMT can be combined with pharmacologic, dietary, and psychological treatments to target somatic contributors to nociceptive facilitation, modulate autonomic tone, reduce reliance on medications, and restore function.

Despite interest in OMT for pain management, reports describing its use in pediatric chronic pancreatitis-associated visceral hyperalgesia remain limited, and its potential role within multimodal pain management strategies is not well defined. The present case describes the use of OMT in a 17-year-old male with acute-on-chronic pancreatitis in the setting of a heterozygous SPINK1 N34S (c.101A > G; p.Asn34Ser) variant who experienced a prolonged hospitalization due to persistent pain and significant meal-related anxiety. This report describes short-term pain improvement associated with OMT within the context of multimodal therapy. The case highlights OMT's potential as an integrative tool in the multimodal management of chronic pancreatitis-associated visceral pain and supports further investigation of this approach.

## 2 Case description

A 17-year-old male with chronic pancreatitis in the setting of a heterozygous SPINK1 N34S (c.101A > G; p.Asn34Ser) variant and obesity presented with recurrent, severe abdominal pain beginning at age 10, with multiple prior hospitalizations for acute exacerbations requiring high doses of opioid medications to manage his pain. He was prescribed pancreatic enzyme replacement therapy for severe pancreatic exocrine insufficiency (most recent fecal pancreatic elastase 10 μg/g), although adherence was poor. During the current admission, he reported right-upper-quadrant and epigastric pain that worsened after meals and produced significant anxiety around eating, resulting in a single-meal-per-day pattern. On presentation his vitals were notable for BP 132/70 mmHg, heart rate 58 bpm, temperature 97.5°F (36.4 °C), respiratory rate 20, and SpO₂ 100%; notably, despite reporting severe pain rated 10/10, he remained bradycardic with a heart rate of 58 bpm, although the clinical significance of this finding is uncertain. In terms of anthropometrics, his weight was 141 kg, and his Body Mass Index (BMI) was 45.43 kg/m². He met institutional criteria for moderate malnutrition based on inadequate nutritional intake, because estimated nutritional intake was approximately 26%–50% of calculated energy requirements. Laboratory evaluation showed a markedly elevated lipase (979 U/L), mild hyperglycemia with normal hemoglobin A1C, mild anemia, and low HDL (18 mg/dL), notably his triglycerides were normal (124 mg/dL). The patient was admitted for acute on chronic pancreatitis the previous month, during which his lipase trended from 471 U/L to 284 U/L. A secretin-enhanced magnetic resonance cholangiopancreatography (MRCP) performed during the prior admission demonstrated calcifications, edema, peripancreatic fat stranding, small intra- and peripancreatic fluid collections, diminished secretin response, splenomegaly with a tortuous splenic vein, focal portal vein narrowing, and hepatic steatosis. Because this imaging had been obtained one month earlier, repeat imaging was not performed during the current hospitalization. On examination he had generalized abdominal tenderness.

Based on his elevated lipase, imaging findings of chronic pancreatic changes, and known genetic predisposition, he was diagnosed with acute-on-chronic pancreatitis. Alternative etiologies for his abdominal pain were considered, including functional abdominal pain related to disorders of gut–brain interaction, complications of chronic pancreatitis such as pseudocyst formation or ductal obstruction, and other intra-abdominal pathology. However, imaging did not demonstrate evidence of pseudocyst formation or ductal obstruction. The combination of markedly elevated lipase, imaging findings consistent with active pancreatic inflammation, and a known SPINK1-associated genetic susceptibility supported acute-on-chronic pancreatitis as the primary driver of symptoms. The persistence and severity of pain disproportionate to objective findings, along with opioid escalation and meal-related anxiety, were most consistent with secondary visceral hyperalgesia and central sensitization in the setting of chronic pancreatic disease. His lipase trended down to 353 U/L by hospital day 2 and 90 U/L by hospital day 3. His course was complicated by prolonged abdominal pain and poor appetite secondary to visceral hyperalgesia. He remained hospitalized for six days during this episode. Table 1 provides a timeline for the patient's hospital course.

**Table 1:** Timeline of inpatient and post-discharge interventions with corresponding pain responses in a 17-year-old male with acute-on-chronic pancreatitis.

Hospital/ post hospital day	Lipase	Daily morphine milligram equivalents	Intervention	Pain score/response
0	923	42	Opioids	Pain 10/10
1	979	75	Opioids	Pain 10/10
2	353	68	Opioids	Pain 10/10
3	90	110	Opioids Considered PCA Started Gabapentin Started Nortriptyline **OMT Session #1**	Pain 6/10 Pain after OMT: 4/10
4	Not performed	32	Opioids Continued Gabapentin Continued Nortriptyline **OMT Session #2**	Pain 3/10 Pain after OMT: 0/10
5	Not performed	7	Opioids Continued Gabapentin Continued Nortriptyline	Not documented
6 (date of discharge)	Not performed	11	Opioids Continued Gabapentin Continued Nortriptyline	Pain 0/10
∼30 (Post Hospital)	Not performed	Not applicable	Continued Gabapentin Continued Nortriptyline **OMT Session #3**	Pain 0/10 Pain after OMT: 0/10
∼100 (Post Hospital)	Not performed	Not applicable	Continued Gabapentin Continued Nortriptyline **OMT Session #4**	Pain 4/10 Pain after OMT: 0/10

The table summarizes hospital and post-hospital days, daily opioid exposure expressed as morphine milligram equivalents (MME), lipase levels, pharmacologic therapy, osteopathic manipulative treatment (OMT) sessions, and associated changes in reported pain scores. OMT sessions (bolded) were followed by immediate reductions in pain during both inpatient and outpatient encounters, while gabapentin and nortriptyline were continued throughout.

Pain control initially required escalating opioid therapy, with daily opioid exposure increasing from 42 morphine milligram equivalents (MME) on admission to a peak of 110 MME on hospital day 3. Because pain remained inadequately controlled, initiation of a morphine patient controlled analgesia (PCA) pump was considered. Despite the increasing opioid requirements, endoscopic intervention was not pursued because prior MRCP did not demonstrate ductal obstruction. Celiac plexus block, ketamine infusion, and inpatient psychological consultation were not utilized during this admission. Instead, because visceral hyperalgesia was suspected, a multimodal regimen was implemented that included gabapentin, nortriptyline, and OMT. OMT was performed by the pediatric gastroenterology fellow, who holds a Doctor of Osteopathic Medicine (DO) degree.

The patient received two inpatient OMT sessions, each lasting approximately 30 min. Treatments were individualized based on somatic dysfunction identified on pre-treatment osteopathic structural examination. The patient was positioned supine for most techniques. Pre-OMT examination identified tissue texture changes and rotational restrictions in the regions corresponding to the celiac, superior mesenteric, and inferior mesenteric ganglia. Techniques were performed in the following sequence: sacral inhibition (approximately 1-2 min of gentle sustained anterior pressure to the sacrum), diaphragmatic doming (approximately 1-2 min of bilateral cephalad traction at the costal margins to improve diaphragmatic excursion), mesenteric release (approximately 4-6 min of direct fascial techniques targeting the celiac, superior mesenteric, and inferior mesenteric ganglion regions), colonic stimulation (approximately 2–4 min of gentle manual stimulation following the anatomical course of the colon), and pedal pump (approximately 1-2 min of rhythmic oscillatory motion to augment lymphatic and circulatory flow). The remaining session time was devoted to osteopathic structural assessment, reassessment of tissue texture and motion restrictions, patient positioning, and transition between techniques. Detailed descriptions of these techniques can be found in standard osteopathic references (8). Pain scores were assessed using a numeric rating scale (0-10) and were recorded 5 min before and 5 min after each session. There was no repeat reassessment. After the first session, pain decreased from 6/10 to 4/10. The second session the following day produced reduction from 3/10 to 0/10. No immediate adverse events were reported. Subsequently his opioid requirements declined to 32.5 MME on hospital day 4, 7.5 MME on hospital day 5, and 11.25 MME on hospital day 6, the day of discharge. On discharge his pain remained controlled, and he was prescribed oxycodone 5 mg as needed for breakthrough pain. In addition to pain reduction, clinical outcomes included opioid discontinuation, improved oral intake, and functional recovery as reflected in reduced meal-related anxiety. Additionally, outpatient psychological follow-up was arranged after discharge.

Post-discharge he continued gabapentin 100 mg nightly and nortriptyline 20 mg nightly with as needed naproxen or acetaminophen; he used only two 5 mg oxycodone tablets over the month after discharge and managed breakthrough discomfort with distraction and postural strategies. The gabapentin 100 mg nightly and nortriptyline 20 mg were kept at these doses being that the pain was overall controlled. At gastroenterology follow-up one month later he reported no pain (0/10). A third OMT session at that visit again documented tissue texture changes and rotational restrictions at the celiac, superior mesenteric, and inferior mesenteric ganglia; treatment included mesenteric release of all three ganglia, balanced ligamentous tension and myofascial release of the gallbladder (approximately 1-2 min of applying gentle, sustained manual pressure to fascial restrictions in the right upper quadrant), colonic stimulation, and pedal pump.

At three-month follow-up, he reported mild to moderate pain (4/10) occurring several times per week. A fourth OMT session documented similar somatic findings at the abdominal autonomic ganglia. Treatment included mesenteric release of all three ganglia, balanced ligamentous tension and myofascial release of the gallbladder, colonic stimulation, anterior thoracic counterstrain (AT9–12), and pedal pump. Pain again decreased immediately from 4/10 to 0/10.

There were no adverse or unanticipated events associated with any of the treatments. OMT effectiveness and tolerability were assessed during each session through direct patient questioning. At multiple points during treatment, the clinician asked the patient to report any changes in pain intensity, abdominal or visceral tension, ease of breathing, areas of discomfort, or new sensations. Non–high-velocity low-amplitude osteopathic techniques (e.g., myofascial release, muscle energy, counterstrain, soft tissue, as used in this case) are generally considered to have a favorable safety profile, with adverse events typically mild and self-limited (9).

Despite improvement in pain following this hospitalization, the patient continued to experience chronic pain. Because of persistent symptoms despite medical management, the family sought a second opinion at a tertiary pediatric pancreas center where total pancreatectomy with islet autotransplantation was recommended and subsequently performed ten months after this episode. He has not had any further episodes of pancreatitis, and his pain is better controlled.

This report was prepared in accordance with the CARE reporting guideline.

## 3 Discussion

Although pain is common during the course of chronic pancreatitis, a subset develop pain phenotypes characterized by abnormal pain processing, central sensitization, and secondary visceral hyperalgesia that may persist independently of acute inflammatory episodes (1, 2). In this case, the patient's pain was consistent with secondary visceral hyperalgesia and central sensitization in the setting of long-standing chronic pancreatitis and a heterozygous SPINK1 N34S variant. However, in interpreting this case, it is important to recognize that the patient's improvement occurred during a resolving episode of acute-on-chronic pancreatitis. Lipase values decreased from 979 U/L on admission to 90 U/L by hospital day 3, indicating biochemical improvement of the acute inflammatory process. Furthermore, gabapentin, nortriptyline, and OMT were all initiated on hospital day 3. Because these interventions occurred simultaneously, the relative contribution of each therapy cannot be determined. The continued decline in pain scores between OMT sessions further suggests that natural resolution of the acute flare and neuromodulator initiation may have contributed substantially to the observed clinical improvement.

The mechanism for pain in the setting of chronic pancreatitis is persistent peripheral inflammation that reshapes nociceptive pathways. Ongoing pancreatic inflammation upregulates proinflammatory and pronociceptive signaling, which sensitizes pancreatic afferent nerves and amplifies pain transmission. Over time, repeated nociceptive input drives central sensitization, marked by exaggerated spinal and cortical pain responses, impaired descending inhibition, and altered pain modulation. These neuroplastic changes lead to visceral pain that is disproportionate to the extent of pancreatic disease and may persist even when local inflammation stabilizes or objective markers improve (1). As a result, patients may experience amplified visceral pain and, in some cases, generalized hyperalgesia severe enough to require high-dose opioid therapy (1, 2).

A complication of this is opioid tolerance and opioid-induced hyperalgesia. This can create a situation where a patient may require increasing higher doses for adequate analgesia, or worse, a paradoxical increase in pain with opioid administration (10). Both of these phenomena can contribute to poorly controlled pain, and may have contributed to this patient's clinical presentation. Licciardone et al. reviewed the potential role of OMT in chronic pain management but did not provide direct evidence of opioid-sparing efficacy (11). More recently, Galeazzi et al. conducted a single-blind, randomized, placebo-controlled trial involving 75 adults receiving palliative cancer care and found that patients treated with OMT experienced greater reductions in pain scores beginning on day 3 of treatment. In a subgroup of patients using patient-controlled analgesia pumps, analgesic demand decreased by 31.6% compared with controls (*P* = 0.016) (12). However, these findings were observed in an adult palliative oncology population and may not be generalizable to pediatric patients with chronic pancreatitis. With our case in mind, more research is needed to determine whether OMT can be seen as a promising non-pharmacologic strategy to mitigate the cycle of escalating opioid requirements.

Given the role of autonomic dysregulation in visceral pain, OMT may provide a mechanism-informed therapeutic approach. By identifying tissue texture changes and modulating viscero-somatic reflexes, OMT has been hypothesized to influence autonomic output, particularly by reducing sympathetic tone, thereby decreasing nociceptive facilitation and supporting improved pain control (7). Importantly, the celiac and superior mesenteric ganglia serve as the primary relay stations for pancreatic sympathetic innervation, housing the postganglionic neurons that directly modulate pancreatic function (13). This anatomical organization reinforces the hypothesis for using targeted OMT techniques in this region. In our case, OMT was applied to address hypothesized autonomic contributors to pain amplification arising from chronic pancreatic inflammation. However, these mechanisms remain hypothetical in this case because no autonomic measurements such as heart-rate variability, catecholamine levels, or skin conductance were obtained.

An important aspect of this case is that the patient ultimately underwent total pancreatectomy with islet autotransplantation (TPIAT) approximately ten months after the index hospitalization. This highlights the distinction between short-term symptom management and long-term disease modification. While OMT was temporally associated with immediate reductions in pain intensity and reduced opioid use during hospitalization, there is no evidence that it altered the underlying progression of chronic pancreatitis or prevented the need for definitive surgical intervention. Rather, the observed benefit may have been limited to symptom management within a broader multidisciplinary treatment strategy. The decision to pursue TPIAT followed persistent chronic pain despite medical management and evaluation at a tertiary pediatric pancreas center. TPIAT remains an important option for carefully selected pediatric patients with refractory chronic pancreatitis and debilitating pain (4).

This case report has several inherent limitations. The most significant limitation was that this was a single-patient observation, and therefore it cannot establish causality. The patient's improvement occurred during a resolving episode of acute pancreatitis, with falling lipase values and concurrent initiation of gabapentin and nortriptyline, making natural disease resolution and neuromodulator therapy important competing explanations. Additionally, documented pain scores decreased from 10/10 on admission to 6/10 before the first OMT session and before initiation of gabapentin and nortriptyline, suggesting that spontaneous clinical improvement may also have contributed to the observed pain trajectory. Pain assessments were based on direct questioning and subjective self-report without validated functional or autonomic outcome measures, were obtained by the treating clinician, who was also an author, and were recorded only 5 min after treatment without independent assessment, blinding, or delayed follow-up measurements, increasing susceptibility to expectation effects and reporting bias. Similarly, the patient perspective was collected by the treating clinician and represents an unblinded self-report, which may be subject to expectation bias. Moreover, exam findings such as tissue texture changes are operator-dependent, which may limit reproducibility and generalizability. Additionally, OMT was performed by a clinician with specialized training, which may limit generalizability to settings without similar expertise. Finally, the patient ultimately underwent TPIAT, indicating that no durable disease-modifying benefit can be inferred from this observation. In terms of adverse events, while OMT is generally considered to have a favorable safety profile, standardized assessment tools and delayed post-session follow-up (e.g., 48–72 h) were not used, which may limit detection of less common or delayed treatment-related effects.

## 4 Conclusion

This case describes a temporal association between OMT and short-term improvement in pain scores within a multimodal treatment strategy for chronic pancreatitis-associated visceral pain. However, concurrent neuromodulator initiation, recovery from an acute inflammatory flare, and the absence of blinded assessment prevent attribution of benefit to OMT alone. While this observation supports further investigation of OMT as a potential adjunctive therapy, no conclusions regarding efficacy or disease modification can be drawn from a single uncontrolled case. Future work should establish a stronger evidence base for OMT across a wider range of visceral pain disorders. Priorities include randomized controlled trials to confirm efficacy and reproducibility across diverse pediatric and adult populations; mechanistic studies to elucidate effects on autonomic balance, visceral hypersensitivity, and gut–brain signaling; and trials evaluating opioid-sparing outcomes. Future studies should incorporate objective physiologic measures, such as heart rate variability and blood pressure, as proxies for autonomic modulation and potential mechanistic effects of OMT. Ultimately, given its low risk, low cost, and bedside applicability, OMT represents a potential adjunct in the management of chronic pancreatitis-associated visceral pain.

## Patient perspective

The patient reported that OMT was one of the first interventions during his hospitalization that provided noticeable and immediate relief. He described feeling “lighter” and more comfortable within minutes of each treatment session, with decreased abdominal tension and improved ability to take deep breaths. He expressed that the hands-on approach felt calming and reassuring, particularly during periods of high anxiety around eating. After discharge, he noted that OMT sessions continued to bring rapid improvement in his symptoms, with reductions in pain that he could “feel right away.” Overall, he felt that OMT played a meaningful role in helping him regain a sense of control over his pain and contributed significantly to his confidence in managing symptoms during recovery.

## Data Availability

The original contributions presented in the study are included in the article, further inquiries can be directed to the corresponding author.
